# The molecular mechanism by which heat stress during the grain filling period inhibits maize grain filling and reduces yield

**DOI:** 10.3389/fpls.2024.1533527

**Published:** 2025-01-17

**Authors:** Xiaohu Li, Shilin Zhuge, Jiyuan Du, Peng Zhang, Xingyu Wang, Tianjian Liu, Donghui Li, Haoran Ma, Xinzheng Li, Yongxin Nie, Changjian Liao, Haiping Ding, Zhiming Zhang

**Affiliations:** ^1^ National Key Laboratory of Wheat Breeding, College of Life Sciences, Shandong Agricultural University, Taian, China; ^2^ Institute of Crops Research, Fujian Academy of Agricultural Sciences, Fuzhou, China

**Keywords:** heat stress, maize, grain filling, kernel development, stress response

## Abstract

High temperatures significantly impair plant growth and development by restricting maize grain filling; however, the molecular mechanisms underlying heat stress remain poorly understood. In this study, 350 maize inbred lines were evaluated under field conditions, leading to the identification of heat-tolerant Zheng58 and heat-sensitive Qi319. The two inbred lines were exposed to controlled conditions of 30°C/20°C (optimal) and 42°C/30°C (heat stress) during the grain filling period. Heat stress significantly reduced thousand-kernel weight and seed setting rates, with Qi319 experiencing more pronounced declines. In contrast, Zheng58 showed superior performance, with a grain filling rate 48% higher and seed setting rate 57% greater than Qi319. Transcriptome analysis showed that heat stress disrupted starch biosynthesis and hormonal homeostasis, notably affecting abscisic acid and auxin pathways. Additionally, photosynthetic and transpiration rates in panicle leaves were reduced due to the downregulation of genes related to light-harvesting complexes, photosystem I subunits, and water transport. These findings highlight the critical roles of starch metabolism, hormonal regulation, and photosynthetic efficiency in heat tolerance, offering valuable insights for developing heat-resilient maize varieties to mitigate yield losses under high-temperature conditions.

## Introduction

Maize (*Zea mays* L.) is a staple food crop and a crucial source of feed and energy. Ensuring its high yield is vital for food security, promoting economic development, and addressing energy crises (Wang et al., 2020; Zhang et al., 2023). Rising temperatures resulting from climate change have significantly affected maize growth, development, geographic distribution, and overall maize quality and productivity (Luo et al., 2023; Li et al., 2024; Niu et al., 2024). Climate warming is anticipated to cause a significant decline in corn yields. Specifically, a 1°C increase in temperature may result in a 6.0% decrease in wheat yield, a 3.2% decrease in rice yield, a 7.4% decrease in maize yield, and a 3.1% decrease in soybean yield (Ray et al., 2019; Agnolucci et al., 2020; Li et al., 2022a; Riedesel et al., 2023). Among these crops, the reduction in maize yield is the most pronounced. Consequently, there is an urgent need to develop heat-resistant maize varieties, and it is crucial to achieve a comprehensive understanding of the effects of heat stress on maize grain filling.

Maize requires an optimal daytime temperature range of 28-32°C for growth, which is higher than that of wheat (*Triticum aestivum*) and rice (*Oryza sativa*) (Sánchez et al., 2014; Harris et al., 2023). In recent years, high temperature weather has occurred frequently around the world, which has had a significant impact on maize production (Niu et al., 2024). Heat stress during the early stages of maize growth can significantly lead to issues such as reduced germination rates and plant wilting (Dutra et al., 2015). During the silk emergence and shedding pollen stages of maize, elevated temperatures can inhibit filament elongation and cause pollen abortion, consequently reducing fertilization and seed-setting rates (Waqas et al., 2021; Gong et al., 2024). Furthermore, heat stress adversely affects the maize pollen shedding rate and vitality, which subsequently impedes grain filling (Lv et al., 2024; Zhang et al., 2024). However, the impact of high temperature on maize grain filling remains unclear.

To adapt heat stress, many factors, including transcription factors, receptor proteins, protein kinases, and metabolic enzymes, function in heat tolerance. Various heat shock factors (HSFs) are induced under high temperature conditions to minimize water loss and prevent plant wilting (Xie et al., 2022; Li et al., 2024). For example, the transcription factor *ZmHsf20* has been identified as a protective agent against heat stress, as it contributes to cell wall remodeling, particularly through its interaction with the cellulose synthase promoter (Li et al., 2024). *ZmbZIP60* enhances the heat tolerance of maize seedlings by upregulating heat-responsive genes, such as *ZmHug1*, which subsequently affects the stability of proteins in the endoplasmic reticulum (Li et al., 2020; Xie et al., 2022). In maize, the transcription factor Necrotic Upper Tips1 (NUT1) plays a crucial role in the response to heat stress by directly participating in the biosynthesis of cellulose during protoxylem development (Dong et al., 2020). Heat stress results in a reduction in pollen grain viability, potentially leading to significant losses. However, the overexpression of the *ZmHsp101* gene allows plants to produce a considerable amount of pollen even under heat stress conditions (Li et al., 2022b). The mitogen-activated protein kinase ZmMPK20 is phosphorylated by its upstream kinase, MAPK kinase 9, thereby enhancing the heat tolerance of maize flowers (Cheng et al., 2023). The impact of heat stress on maize yield is contingent upon the concurrent occurrence of heat stress and the maize flowering period. While numerous studies have confirmed that heat stress significantly affects male tassels, the potential molecular regulators of high temperature’s effects on female flowers and grain filling remain to be elucidated.

Grain yield decreases when grains are filled under heat stress. Endosperm endogenous processes influence the rate and duration of dry weight gain (Wang et al., 2024). The grain filling involves the synthesis and accumulation of starch within the grain. It is widely accepted that maternal nutrients, including sucrose, are transported to the seeds (Yan et al., 2019; Shirdelmoghanloo et al., 2022). The nutrient transport capacity of transfer cells, along with the growth rate of grains, is critical for the grain filling process. This process is regulated by hormones, photosynthesis, and various environmental factors (Cui et al., 2020; Yu et al., 2024). During the grain filling period of maize, heat and drought stress are commonly experienced. Heat stress accelerates the grain filling process while simultaneously reducing starch content (Wu et al., 2024b). This stress negatively impacts organ development and significantly affects grain filling (Qiao et al., 2023). Heat stress inhibits the enzymatic synthesis of starch, thereby hindering the accumulation of essential grain components, and also decreases the activity of starch synthesis (Qu et al., 2023). Specifically, the activities of adenosine diphosphate-glucose pyrophosphorylase, soluble starch synthase, and starch branching enzymes are diminished, leading to abnormal grain filling. Furthermore, heat stress during the filling period increases the average size of starch granules and the proportion of long chains in amylopectin. These alterations affect the structural characteristics of maize starch, influencing its expansion and thermal properties (Lu et al., 2014; Qu et al., 2023).

High temperatures can diminish photosynthetic efficiency, resulting in shorter life cycles and reduced crop yields (Gabaldón-Leal et al., 2016; Ribeiro et al., 2020; Wang et al., 2023). Furthermore, elevated temperatures lead to a decline in respiratory enzyme activity, which causes energy deficiencies. Additionally, the heat-induced movement of biomolecules disrupts plasma membrane homeostasis (Li et al., 2022c). However, the mechanisms by which heat stress affects maize grain filling remain unclear. Currently, there are limited studies addressing the effects of high temperatures on maize grain filling. This paper compares the grain filling rate (GFR), thousand-kernel weight (TKW), seed setting rate and pollen activity of the heat-resistant inbred line Zheng58 and the heat-sensitive inbred line Qi319, determining that the impact of high temperature on their grain filling is not attributable to pollen. By exploring the differences in gene regulation between the two inbred lines, we clarified the variations in endosperm development, sugar transport, starch synthesis, hormone levels, photosynthesis, and transpiration. Our results enhance the conceptual understanding of the seed setting rate’s response to heat stress during the grain filling period of maize and investigate the intrinsic regulatory network, providing important information for breeding new heat-tolerant maize varieties.

## Materials and methods

### Plant materials and experimental design

In the summer of 2021, a total of 350 distinct maize inbred lines, encompassing both local and elite varieties from temperate, subtropical, and tropical germplasm, were planted in the experimental field of Shandong Agricultural University in Tai’an, China (35.6°N, 117.5°E) (Yang et al., 2011; Li et al., 2023). During this period, the inbred lines suffered severe heat damage and had a low seed setting rate, which prompted us to focus on identifying five heat-tolerant inbred lines, five general inbred lines, and five heat-sensitive inbred lines.

To further verify the heat tolerance phenotype of these 15 maize inbred lines, a randomized complete block design with three replications was employed in the Shandong Agricultural University experimental field located in Tai’an, Shandong, during the year 2022. The heat sensitivity of the 15 maize inbred lines was assessed. Sowing occurred on June 10th, pollination date is around August 15th, with harvest taking place between October 10th and October 15th. Ultimately, the heat-tolerant maize inbred line Zheng58 and the heat-sensitive inbred line Qi319 were identified.

The experiment took place in 2023 in a constant temperature greenhouse at the Shandong Agricultural University Experimental Station. Maize inbred lines Zheng58 and Qi319 were cultivated at a controlled temperature of 30°C/20°C (daytime maximum/nighttime minimum) until pollen maturity. One group of maize was transferred to a higher temperature setting of 42°C/30°C (daytime maximum/nighttime minimum) until harvest, while the other group remained at 30°C/20°C for the entire growth period. Both groups received adequate water and nutrients.

### Measurement for thousand-kernel weight and grain-filling rate

In the field trial, ears from each variety were harvested during the grain filling stage. The kernels were subsequently separated from the cobs, counted, weighed, and dried at 80°C until a constant weight was achieved. To calculate the thousand-kernel weight (TKW), 100 kernels from each plot were counted and weighed post-drying, with three replicates performed. The grain filling rate (GFR), which represents the increase in grain weight from mid-grain filling to maturity, was determined and calculated as previously described. After the application of the pollen dispersal, three representatives, unpollinated, bagged female ears were selected, each with three biological replicates. The number of florets was determined using the same method as for determining the ear grain number. For each female ear, the number of rows per ear and the number of florets per row were counted, and the average was calculated to determine the final number of florets (Ma et al., 2015). The seed setting rate (%) was calculated as follows: (number of grains per spike/number of florets)*×*100%.

### Fluorescein diacetate (FDA) staining

Male tassels of maize were collected from the field, with some samples placed in an incubator set at 42°C. Pollen was harvested for staining after a heat stress treatment lasting 6 hours. The pollen was transferred to a 1.5 ml centrifuge tube, washed once with saline, and treated with 100 μg/ml fluorescein diacetate (FDA). Following a 5-minute incubation in the dark at room temperature, the cells were washed 1-2 times with saline, suspended in distilled water, and prepared for observation via smear. Images of the Alexander-stained and FDA-stained samples were captured under bright light and GFP light, respectively, using a fluorescence microscope (LSM880; Zeiss, Germany). For scanning electron microscopy (SEM), fresh mature pollen grains were directly coated with gold and analyzed using a Zeiss EVO scanning electron microscope (Carl Zeiss Microscopy GmbH, Jena, Germany).

### RNA extraction and real-time quantitative PCR

Total RNA was extracted from the kernels, stems, and leaves of Zheng58 and Qi319 subjected to heat stress was extracted using TRIzol reagent (Invitrogen). First-strand cDNA was synthesized with a quantitative reverse transcription kit (Aikoway) following the manufacturer’s instructions. Reverse transcription quantitative PCR (qRT-PCR) was performed using SYBR Green mix (Quanti Nova SYBR Green PCR Kit, Roche). *ZmActin* and *Zm18S* served as internal controls for normalizing gene expression, and the relevant gene expression data were calculated using the 2^−△△Ct^ method. The analysis included three biological replicates and three technical replicates for each gene. The primer sequences for each gene are provided in **Supplementary Table S1**.

### RNA-seq analysis

Total RNA was extracted from the kernels, leaves, and stems of Zheng58 and Qi319, both before and after heat stress treatment, using TRIzol (Invitrogen). Transcriptome analysis, conducted with CapitaBio Technology, included three biological replicates. Library construction adhered to standard Illumina protocols. Reads were mapped to the maize genome using TopHat2, and genes exhibiting adjusted *P*-values of less than 0.01 were identified as differentially expressed (Gill and Dhillon, 2022; Withanage et al., 2022).

### Determination of carbohydrate, sucrose synthase and hexokinase activities

Kernels, ear leaves, ear stem internodes, and cobs from the thermotolerant inbred line Zheng58 and the thermosensitive inbred line Qi319 were subjected to both control and heat stress treatments. The collected samples were promptly frozen in liquid nitrogen and subsequently analyzed for carbohydrates, soluble sugars, starch, sucrose, glucose, and fructose. The enzyme activities of sucrose synthase and hexokinase were assessed as previously described (Zhao et al., 2015; Shen et al., 2020). To determine starch content, we employed a starch determination kit (BC0700, Solarbio), following the specific procedural guidelines provided. Similarly, for sucrose quantification, we utilized a sucrose determination kit (BC2465, Solarbio), adhering to the detailed instructions outlined in the kit.

### Plant hormone content determination

The kernels 7 days after pollination and stems of Zheng58 and Qi319 before and after high temperature treatment were freeze-dried in liquid nitrogen and stored at -80°C until use. The dried plant material was then homogenized and ground into powder in a grinder. 100 mg of the dried powder was extracted with 1.5 mL of a mixed solution of MeOH: H_2_O: FA (79.9: 20: 0.1). The extract was vortexed and sonicated for 30 minutes and then placed at 4°C for 12 h. The supernatant was collected after centrifugation. The residue was re-extracted with 1 mL of MeOH under sonication for 30 minutes and centrifuged. The supernatants were mixed and then evaporated to dryness under a stream of nitrogen and then reconstituted in 100 μL of a mixed solution of MeOH: H_2_O (50: 50). The solution was filtered through a 0.22 μm filter for further LC-MS analysis. The samples were analyzed using an HPLC-MS/MS system Triple Quadrupole 4500. The HPLC analytical conditions were as follows: column was HYPERSIL GOLD C18 column (3 μm, 2.1 mm*100 mm); solvent A was H_2_O (0.1% FA); solvent B was MeOH; gradient program, 90% A from 0min to 0.2 min, 90% A at 3 min and kept to 8min, 10% A at 8 min and kept to 10 min; flow rate, 0.3 mL/min; temperature, 35°C; injection volume: 5 μL. Triple Quadrupole 4500 HPLC-MS/MS System, equipped with an ESI ion source, was operated in both positive and negative ion mode and controlled by Analyst 1.6.3 software (Sciex). The ESI source operation parameters were as follows: ion source, ESI+/-; ion spray voltage was 5500 V/4500 V, source temperature was 550°C; curtain gas (CUR) was set at 30 psi; the collision gas (CAD) was 9 psi. DP and CE for individual MRM transition was done with further DP and CE optimization. A specific set of MRM transitions were monitored for each period according to the phytohormones eluted within this period.

### Determination of photosynthetic rate, transpiration rate and water content of leaves

The measurements were conducted using the LI-6400 portable photosynthesis system (LI-COR, Lincoln, USA) at a light intensity of 1000 μmol m² s^-1^. Initially, the leaves were excised from the plant and their weight was recorded. Subsequently, the leaves were placed in a controlled environment with constant temperature and humidity, and their weight was measured again after a specified duration. By comparing the weight difference between the two measurements, the amount of water transpired from the leaves can be calculated. Finally, the leaves and seeds were dried at 80°C to determine their water content.

### Statistical analysis

Data were analyzed and plotted using Origin 2021 (Origin Lab), Excel (Microsoft 365), and GraphPad Prism v. 8.0.2. The statistical analyses for all experiments are described in the figure legends. Comparisons between two groups were performed using two-tailed Student’s t-tests. For comparisons between three or more groups, one-way analysis of variance was conducted, followed by Tukey’s honestly significant difference test. *P* values were used to determine the statistical significance, with the following notations: ns, no significant difference (*P* ≥ 0.05); *P < 0.05; **P < 0.01; ***P < 0.001. The panels of micrographs show representative results from one of three independent experiments.

## Result

### High temperatures limit the grain filling rate and reduce grain weight

**Figure 1:**
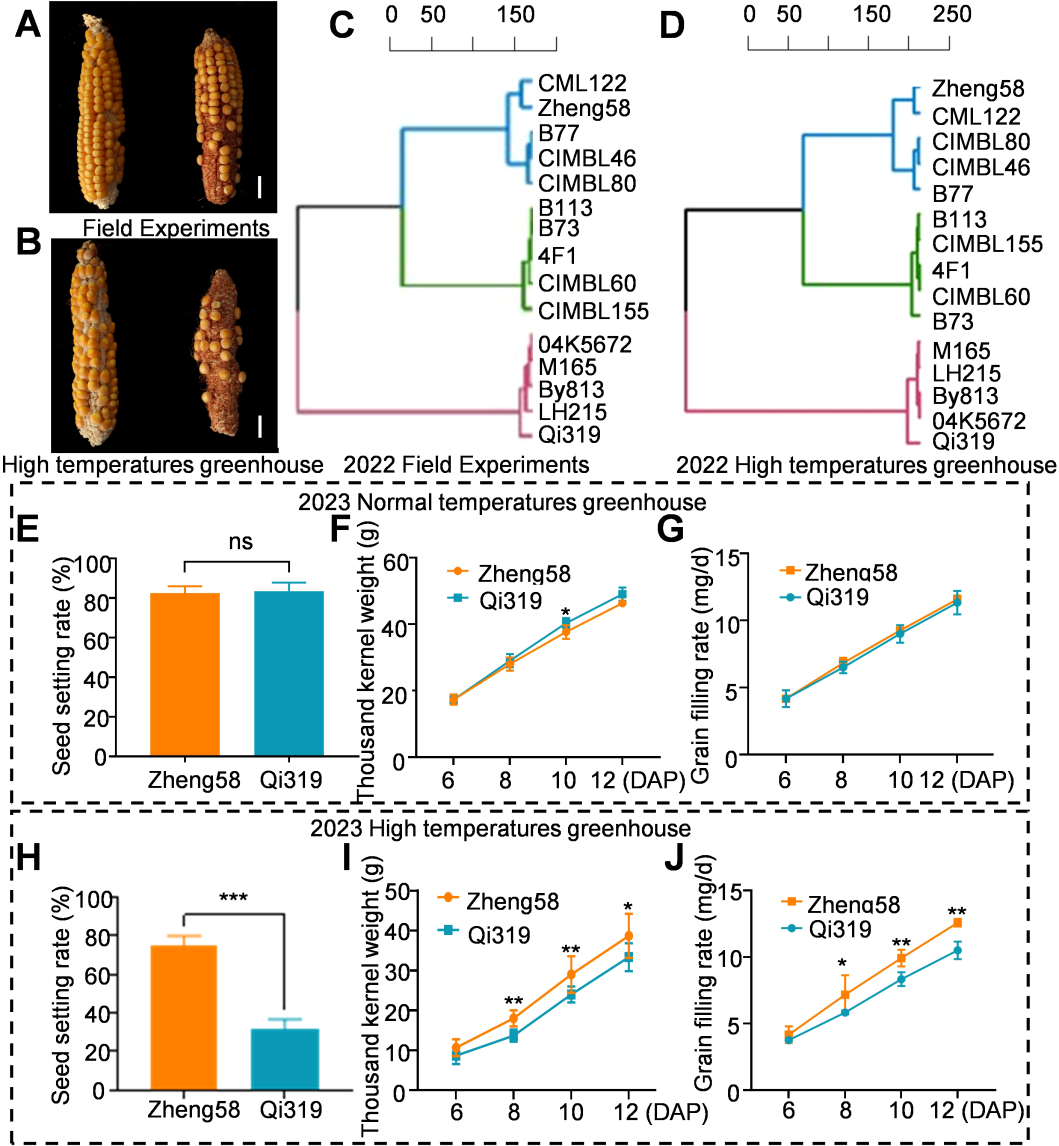
The influence of heat stress on yield traits of Zheng58 and Qi319. **(A, B)** The ear phenotypes of Zheng58 and Qi319 were obtained in the field in 2022 **(A)**, and in the high-temperature greenhouse in 2022 **(B)**. Scale bars = 1 cm. **(C)** Grain filling rate of various maize inbred lines from mid-grain filling to maturity during the 2022 field trial. **(D)** Grain filling rate of different maize inbred lines from mid-grain filling to maturity in the high temperature greenhouse in 2022. **(E–G)** Seed setting rate **(E)**, thousand-kernel weight **(F)** and grain filling rate **(G)** of the normal temperature greenhouse at 6, 8, 10 and 12 days after pollination. Values are shown as the mean ± standard error (SE) from three biological repeats. Statistically significant differences were identified between pairs of measurements using Student’s t-test (*P < 0.05), “ns” denotes no significant difference. **(H–J)** Seed setting rate **(H)**, thousand-kernel weight **(I)** and grain filling rate **(J)** of maize grown in a high-temperature greenhouse at 6, 8, 10, and 12 days after pollination. Values are shown as the mean ± standard error (SE) from three biological repeats. Statistically significant differences were identified between pairs of measurements using Student’s t-test (*P < 0.05, **P < 0.01, ***P < 0.001).

Maize grain filling is a complex quantitative trait influenced by the timing and severity of stress during plant growth and development (Dong et al., 2021). Heat stress significantly impacts the GFR, TKW and seed setting rate (Chen et al., 2024). In 2021 study involving the planting of 350 inbred lines, observed that heat stress significantly impacted the seed setting rate of maize. We identified five maize inbred lines with high seed setting rates, five with low seed setting rates, and five with average seed setting rates. In 2022, we planted 15 inbred lines across multiple locations, including a high-temperature greenhouse. The field seed setting phenotypes of these 15 maize inbred lines were consistent with those observed in the greenhouse. Notably, Zheng58 exhibited a superior seed setting rate, while Qi319 demonstrated a lower seed setting rate (**Figures 1A, B**; **Supplementary Figures S1A, B**, **Supplementary Table S2**). This indicates that the observed differences in seed setting rates between the two inbred lines were influenced by heat stress. From the early filling stage to maturity, both field and greenhouse experiments demonstrated that the inbred line Zheng58 exhibited the highest GFR, while Qi319 displayed the lowest GFR (**Figures 1C, D**; **Supplementary Table S2**).

To better understand the differences between Zheng58 and Qi319, we planted both two inbred lines in a greenhouse in 2023. Following pollination, the growth groups were subjected to heat stress treatments of 42°C/30°C and 30°C/20°C, respectively. Under normal temperature conditions (30°C/20°C), both Zheng58 and Qi319 exhibited similar phenotypes, including seed setting rate, GFR, and TKW. Notably, the grain filling rate of Qi319 surpassed that of Zheng58 (**Figures 1E–G**). However, under heat stress (42°C/30°C), starting from the 6th day after pollination Zheng58 demonstrated significantly higher seed setting rates, grain filling rates, and thousand kernel weights compared to Qi319 (**Figures 1H–J**). Statistical analysis of the seed setting rates for the two inbred lines revealed that heat stress resulted in a 57% reduction in the seed setting rate of the Qi319 inbred line compared to that of the Zheng58 inbred line. These findings indicate that plant responses to heat stress are genotype-dependent, with distinct responses observed among different inbred lines.

**Figure 2:**
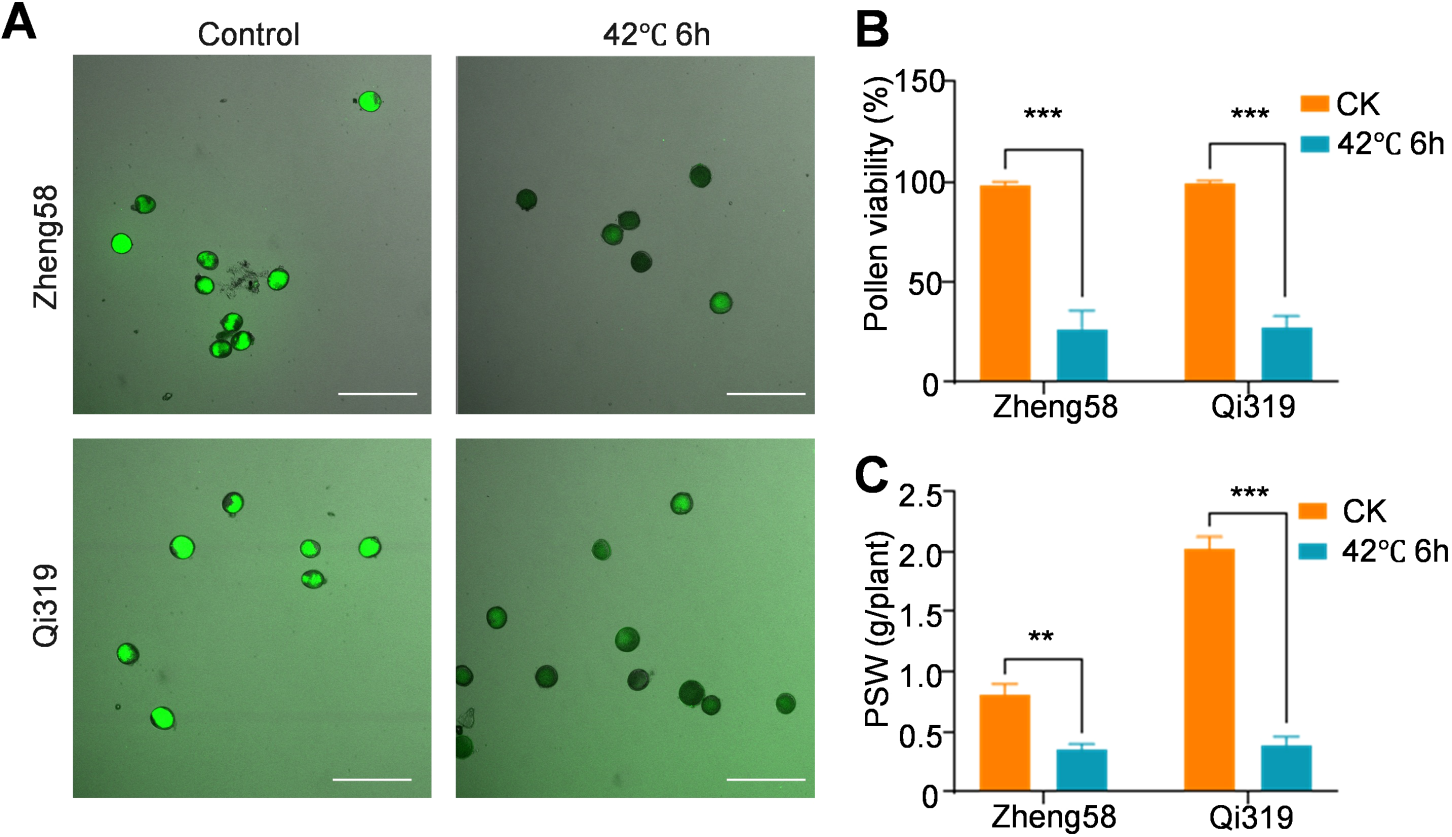
Pollen viability determination after heat stress treatment. **(A)** The pollen viability of inbred lines Zheng58 and Qi319 before and after heat stress treatment was examined by fluorescein diacetate (FDA) staining. The intensity of the fluorescence signal indicated the intensity of pollen activity. Bars= 20 μ0r **(B)** The relative pollen vitality was measured, with the vitality of Zheng 58 under normal conditions as 1. Values are shown as the mean ± standard error (SE) from three biological repeats. Statistically significant differences were identified between pairs of measurements using Student’s t-test (***P < 0.001). **(C)** The pollen shedding weight (PSW) was measured. Values are shown as the mean ± SE from three biological repeats. Statistically significant differences were identified between pairs of measurements using Student’s t-test (**P < 0.01, ***P < 0.001).

Under heat stress, maize faces challenges in flowering, resulting in decreased pollen vitality and inhibited pollen shedding (Begcy et al., 2019). We assessed pollen viability before and after exposure to elevated temperatures, and both FDA staining and pollen viability assays demonstrated that heat stress treatment led to a substantial reduction in pollen viability for both Zheng58 and Qi319; however, no significant differences were noted between the two genotypes (**Figures 2A, B**). Following heat stress treatment, the pollen shedding weight (PSW) decreased by 58% for Zheng58 and 82.8% for Qi319 (**Figure 2C**). Importantly, the PSW of Zheng58 was significantly lower than that of Qi319. At the same time, the expression changes of pollen development and pollen vitality-related genes *ANXUR* (*ZmANX*), the phosphatidylinositol 4-phosphate 5-kinase (*ZmPIP5K*), and serine/threonine kinase (*ZmSTK*) were determined (Boisson-Dernier et al., 2009; Ugalde et al., 2016; Fan et al., 2018). Heat stress treatment caused them to be significantly down-regulated in Zheng 58 and Qi319. However, there was no significant difference between Zheng58 and Qi319 (**Supplementary Figures S2A–C**). In addition, to exclude the impact of pollen viability on yield, we analyzed the expression changes of several representative genes in the silk of Zheng58 and Qi319 following heat stress. After heat stress, the expression levels of basic leucine zipper 25 (*ZmbZIP25*), S-locus glycoprotein (*ZmSLG*), and stigma-specific peroxidase (*ZmSSP*) genes were significantly down-regulated due to heat stress (McInnis et al., 2005, 2006; Li et al., 2018; Sehgal and Singh, 2018); however, no significant differences were observed in the changes between the two inbred lines (**Supplementary Figures S2D, E**). This indicates that the effect of heat stress on the yield of Zheng58 and Qi319 is not solely related to the tassel.

### Pathway enrichment analysis of differentially expressed genes in maize kernels under heat stress

**Figure 3:**
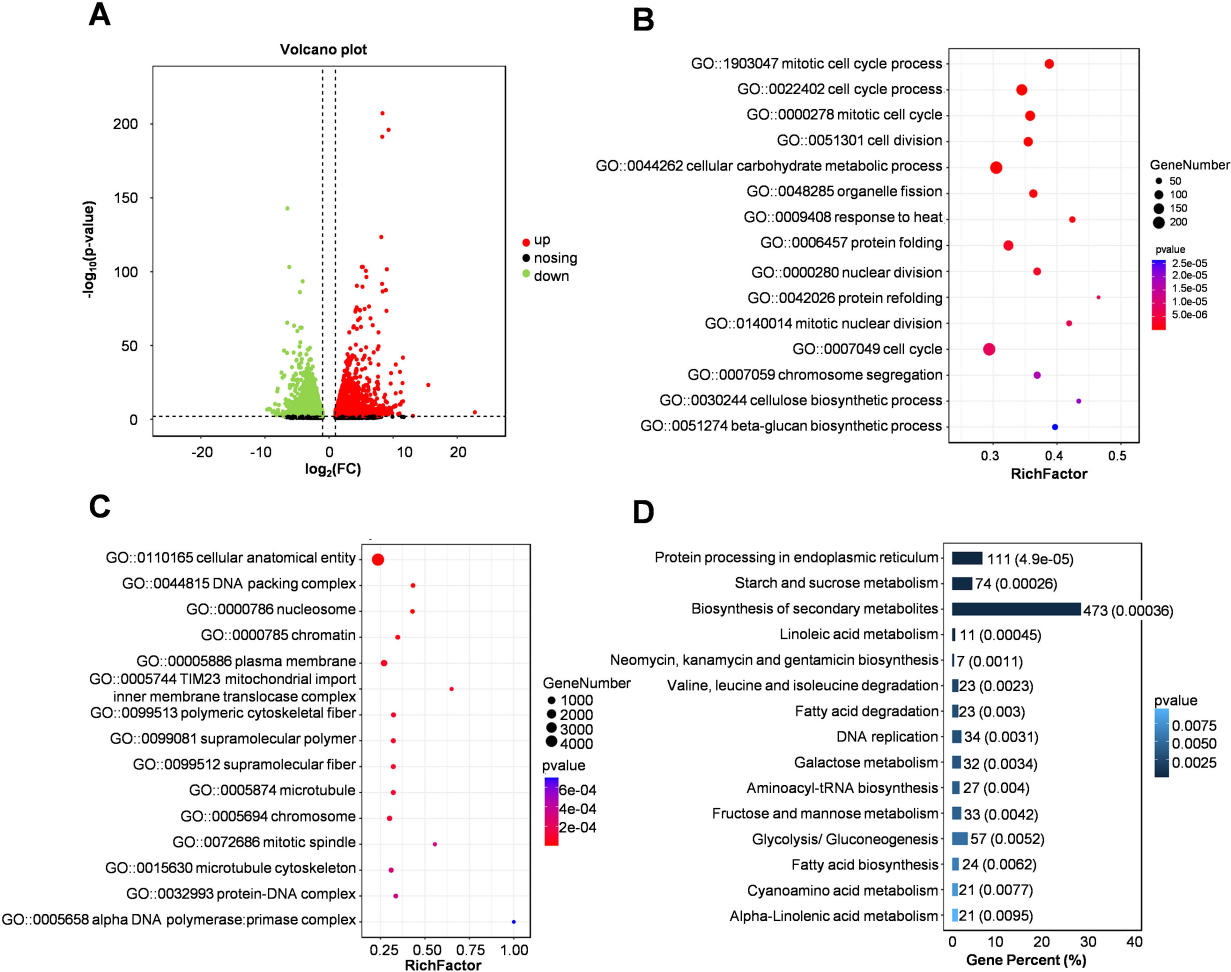
Pathway enrichment analysis of differentially expressed genes in maize kernels subjected to heat stress. **(A)** The scatter plot illustrates gene expression in Zheng58 kernels before and after exposure to heat stress treatment. Red dots indicate up-regulated genes, while green dots denote down-regulated genes. **(B, C)** Gene Ontology (GO) functional enrichment analysis is presented, with the size of the circles reflecting the number of enriched genes. **(D)** Kyoto Encyclopedia of Genes and Genomes (KEGG) pathway enrichment analysis of up-regulated genes is displayed, with the numbers representing both the quantity and proportion of enriched genes.

The inbred line Zheng 58 showed obvious adaptability at the early stage of grain filling, and began to show significant phenotypic differences with Qi319 6-8 days after pollination (**Figures 1E–G**). To analyze the heat-response genes and regulatory networks in maize kernels under heat stress, we conducted RNA-Seq on maize kernels subjected to heat treatment and those without treatment, seven days after pollination. In both the treatment and control groups, we identified a total of 8754 differentially expressed genes (DEGs): 4065 were down-regulated and 4689 were up-regulated (**Figure 3A**). Notably, the gene, embryo surrounding region2 (*ZmESR2*) exhibited significant up-regulation (**Figure 3A**; **Supplementary Table S3**). Further analysis of the Gene Ontology (GO) functional enrichment of these differentially expressed genes (DEGs) revealed their primary involvement in processes such as mitotic cell cycle, cellular carbohydrate metabolism, and response to heat stress (**Figures 3B, C**). Kyoto Encyclopedia of Genes and Genomes (KEGG) pathway analysis indicated that the differentially expressed genes (DEGs) were associated with the biosynthesis of secondary metabolites, as well as sugar and starch metabolism (**Figure 3D**). A comparative analysis of the up-regulated and down-regulated genes showed that the down-regulated genes were primarily involved in ion binding, metabolic pathways, and the biosynthesis of both secondary metabolites and sugars and starch (**Supplementary Figures S3A–C**). The up-regulated genes were predominantly enriched in processes related to protein folding, response to heat and sucrose metabolism (**Supplementary Figures S3D–F**). This suggests that under heat stress, maize primarily maintains homeostasis and reduces cell death as a response. Furthermore, heat stress facilitates the development of embryo-related tissues and enhances plant reproduction. Concurrently, genes associated with growth and development are significantly downregulated, which adversely affects cell division and starch accumulation in maize kernels.

### Enrichment analysis of differentially expressed genes between Zheng58 and Qi319

**Figure 4:**
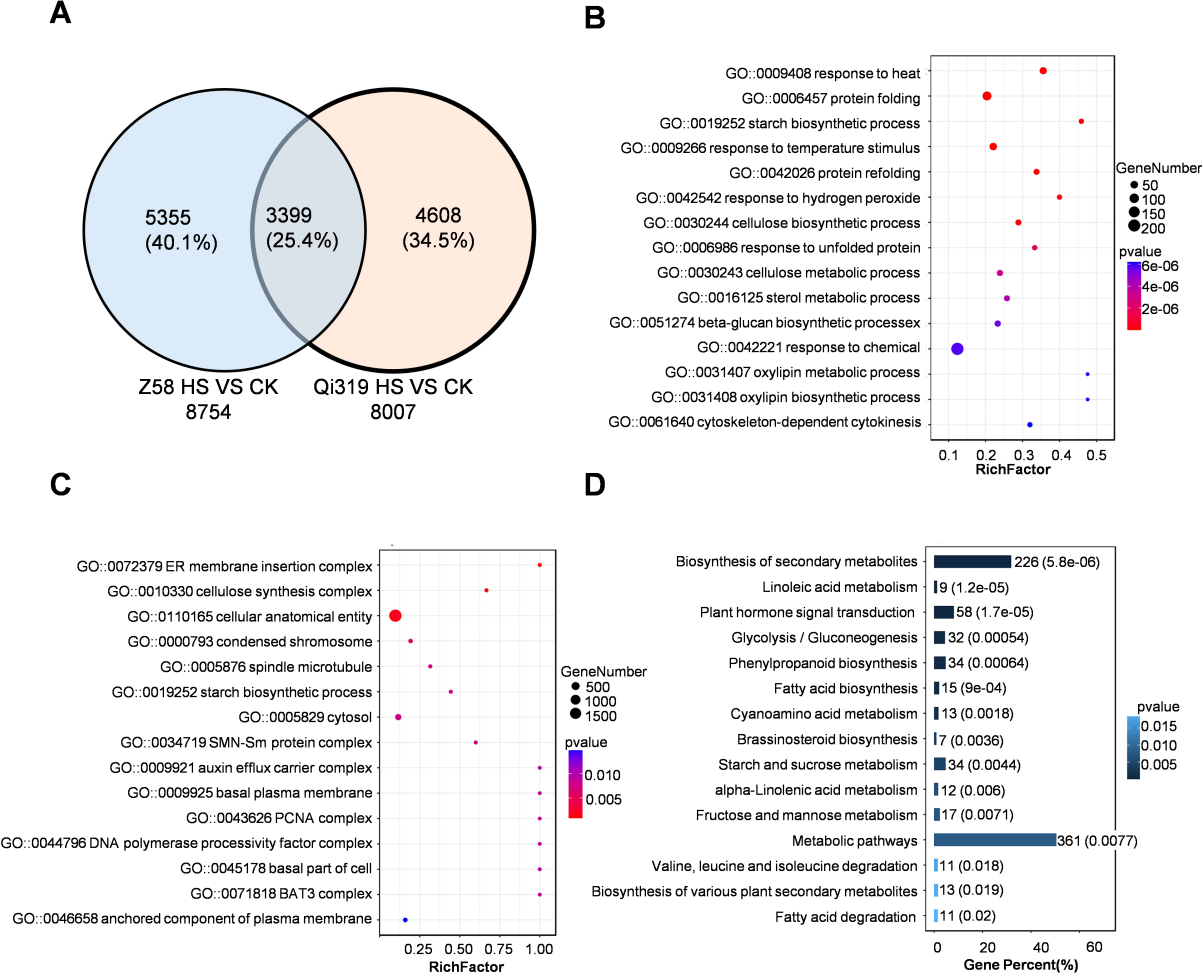
The enrichment analysis of differentially expressed genes (DEGs) in the kernels of Zheng58 and Qi319 is presented. **(A)** A Venn diagram illustrates the DEGs in Zheng58 and Qi319 grains subjected to heat stress. **(B, C)** depict the GO analysis of DEGs that are co-regulated in the kernels of the two maize inbred lines, with **(B)** focusing on biological processes and **(C)** on molecular biological functions. The size of the bubbles corresponds to the number of enriched genes. **(D)** The KEGG analysis of DEGs co-regulated in the kernels of the two maize inbred lines under heat stress is shown, with the numbers indicating both the quantity and proportion of enriched genes.

To investigate the pathways affecting maize grain filling under heat stress, we performed RNA sequencing on grains from the heat-tolerant inbred line Zheng58 and the heat-sensitive line Qi319 after a 7-day heat stress treatment. We identified 8754 and 8007 DEGs in Zheng58 and Qi319, respectively, when compared to control conditions (**Figure 4A**; **Supplementary Table S4**). Under heat stress, the number of differentially expressed genes between the two inbred lines was 3399 (**Figure 4A**). According to gene ontology enrichment analysis for the 3399 DEGs, a significant proportion of these genes were categorized as involved in responses to heat stress and incorrect protein folding (**Figures 4B, C**). KEGG pathway analysis further revealed that these DEGs primarily participated in the biosynthesis of metabolites, metabolism of sugars and starch, and responses hormones (**Figure 4D**). The results demonstrate that heat stress significantly impacts the metabolism of sugars, starches, and hormonal levels in kernels. A further comparison was made between the differentially expressed genes in Zheng58 and Qi319, comparison of differentially expressed genes before and after heat stress treatment revealed 5074 genes, constituting 36.2% of the total gene count (**Supplementary Figure S4A**). GO enrichment analysis showed that the differentially expressed genes were mainly involved in light signal response, sugar starch metabolism, endoplasmic reticulum processing, and other metabolic biosynthesis (**Supplementary Figures 4B–D**). Collectively, these results indicate that Zheng58 and Qi319 exhibit differences in light signal transduction, sugar and starch metabolism, and hormonal responses following heat stress.

### Differential gene expression in maize kernels related to starch and sucrose metabolism under heat stress

**Figure 5:**
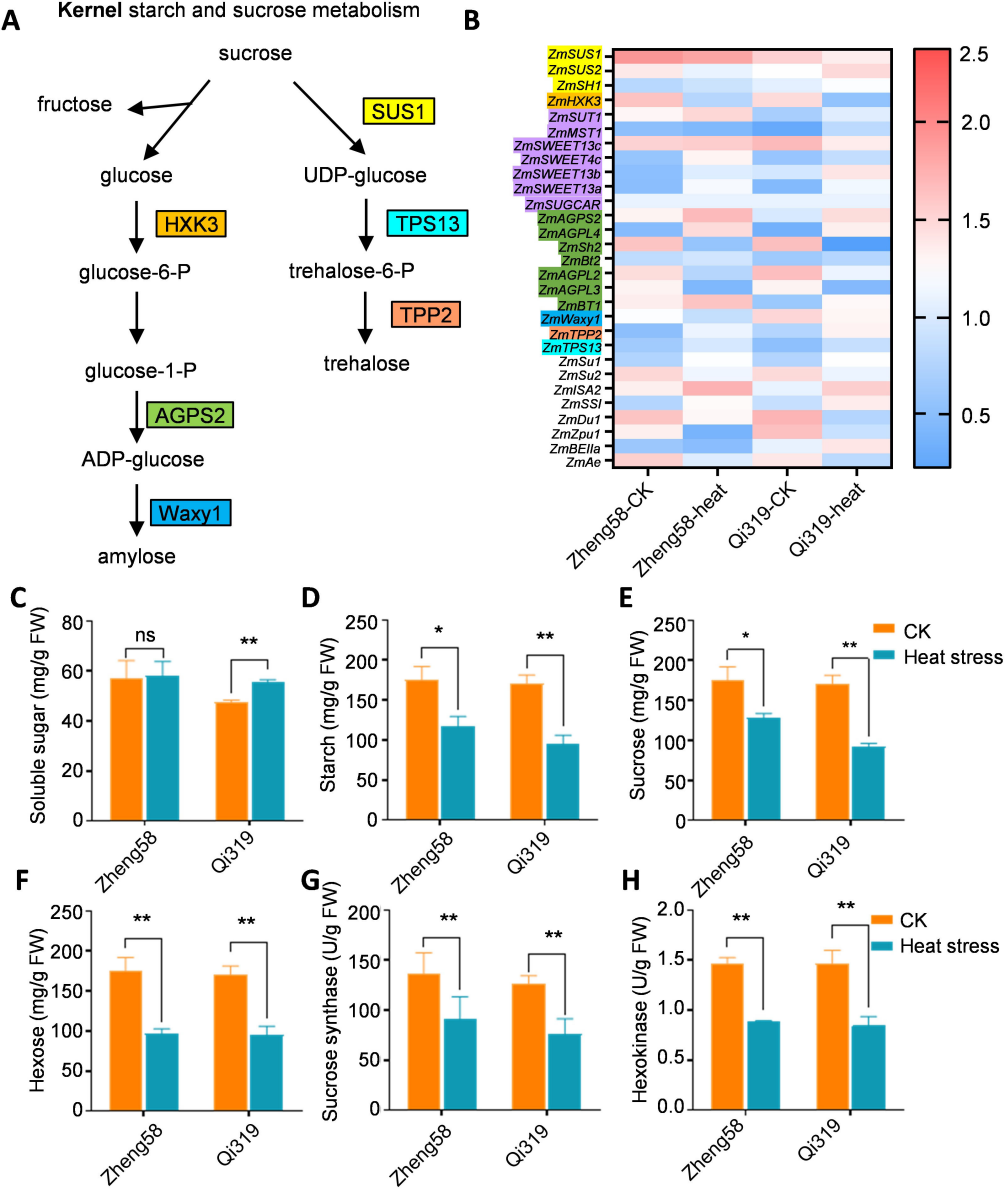
Enrichment analysis of differentially expressed genes involved in starch and sucrose metabolism in maize kernels before and after heat stress treatment. **(A)** Sugar and starch metabolism signaling pathways in maize, with different colors representing different family genes. **(B)** Expression analysis of differentially expressed genes involved in sucrose and starch metabolism in Zheng58 and Qi319 maize kernels before and after heat stress treatment. The value represents the log_2_fold change in the expression level of the gene under different comparisons. Red indicates up-regulated expression, and blue indicates down-regulated expression. **(C–F)** Soluble sugar **(C)**, starch **(D)**, sucrose **(E)**, and hexose **(F)** in Zheng58 and Qi319 before and after heat stress treatment, Values are shown as the mean ± SE from three biological repeats. Statistically significant differences were identified between pairs of measurements using Student’s t-test (*P < 0.05, **P < 0.01), “ns” denotes no significant difference. **(G, H)** Sucrose synthase **(G)** and hexokinase **(H)** enzyme assays in Zheng58 and Qi319 maize kernels under treatment and without treatment. One-way ANOVA with Tukey’s multiple comparison test (*P < 0.05, **P < 0.01).

GO analysis revealed a significant inhibition of genes associated with starch, sucrose, and carbohydrate metabolism under heat stress. To investigate the responses of sucrose and starch metabolism to elevated temperatures, we employed qRT-PCR to assess expression differences. The results indicated a significant reduction in sucrose synthase (*ZmSUS*) expression in Qi319 under heat stress, while in Zheng58, the decrease was not significant (**Figures 5A, B**). Notably, *ZmSUS* expression levels were significantly higher in Zheng58 compared to Qi319. Additionally, heat stress also lowered hexokinase (*ZmHK*) expression in both inbred lines. Under heat stress, Zheng58, the heat-resistant inbred line, showed higher *ZmHK* expression in the grains than Qi319 (**Figures 5A, B**). To further elucidate the impact of heat stress treatment on sugar and starch content in maize kernels, we analyzed the carbohydrate content and enzyme activities of *ZmSUS* and *ZmHK* in two inbred lines under heat stress (**Figures 5C–H**). In both inbred lines, maize kernels exposed to heat stress had higher soluble sugar content and lower starch content compared to untreated kernels (**Figures 5C, D**). Soluble sugars play a crucial role in helping plants resist abiotic stress. Heat stress significantly reduced sucrose concentrations in the grains of both inbred lines, with a greater reduction observed in Qi319 (**Figure 5E**). Conversely, heat stress significantly boosted hexose (glucose and fructose) content in two inbred lines, particularly in Zheng58 (**Figure 5F**), aligning with transcriptional level observations. We also assessed the enzyme activities of sucrose synthase (SUS) and hexokinase (HK). Heat stress inhibited these enzyme activities in both inbred lines, especially in Qi319 (**Figures 5G, H**). The findings revealed reduced gene expression related to sugar and starch metabolism in Qi319, leading to decreased sugar transport and starch synthesis, thereby delaying grain filling.

### Heat stress disrupt hormone homeostasis and impair grain filling

**Figure 6:**
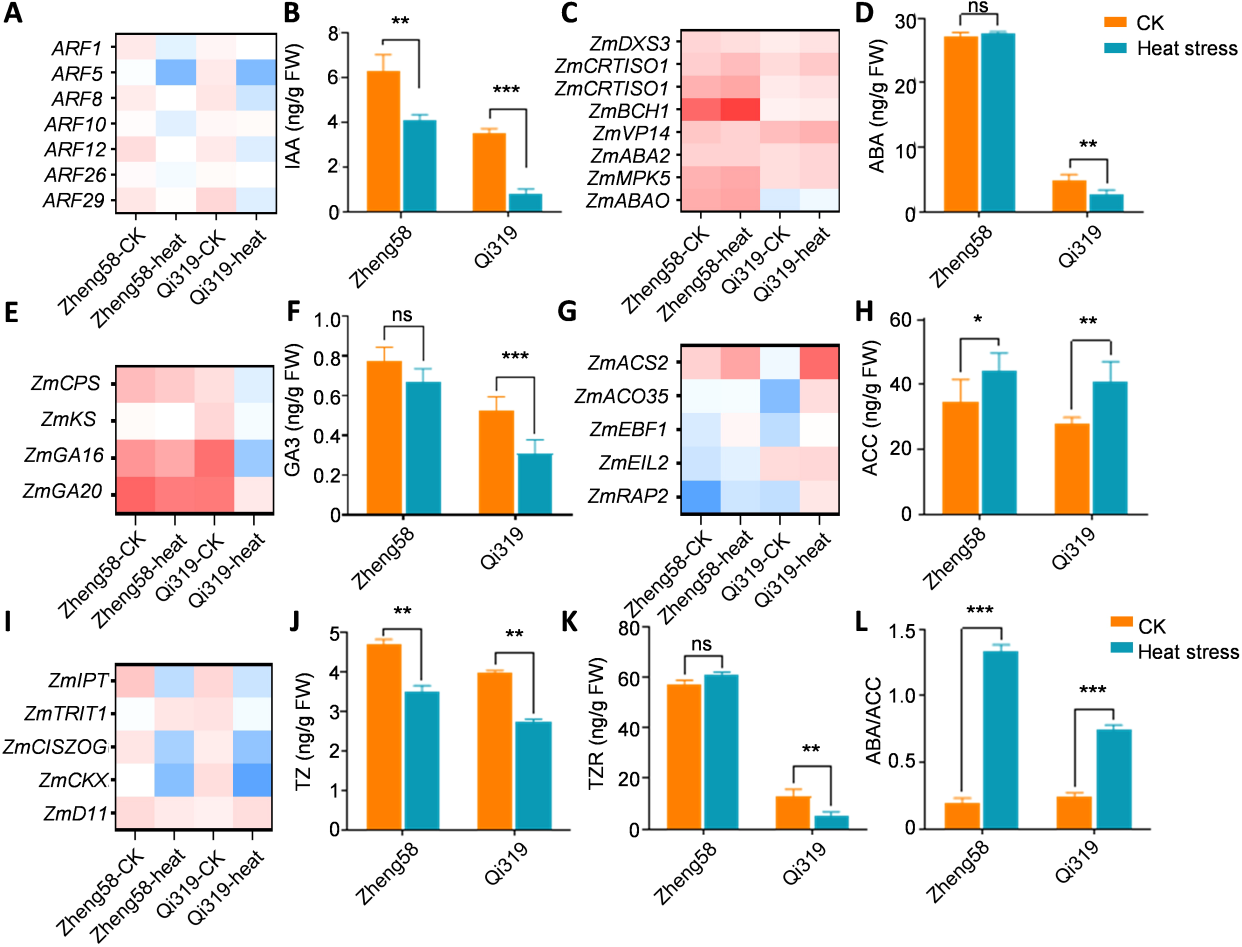
Responses of phytohormones in maize kernels under high temperature conditions. **(A, B)** Differentially expressed genes (DEGs) associated with auxin synthesis and transport **(A)** and auxin content **(B)** in maize kernels under control (CK) and high temperature (HT) conditions. **(C, D)** DEGs related to abscisic acid (ABA) ynthesis and response in maize kernels under CK and HT conditions, with a focus on ABA content **(D)**. **(E, F)** DEGs pertaining to gibberellin (GA) synthesis and response in maize kernels under CK and HT conditions **(E)** and GA3 content **(F)**. **(G, H)** DEGs associated with ethylene synthesis and response **(G)** and the content of the ethylene precursor ACC **(H)** in maize kernels under CK and HT conditions. **(I–K)** DEGs related to cytokinin synthesis and response in maize kernels under CK and HT conditions **(I)**, including the determination of zeatin (TZ) **(J)** and transzeatin riboside (TZR) contents **(K)**. **(L)** The ratio of ABA to ACC is also analyzed. Values are shown as the mean ± standard error (SE) from three biological repeats. Statistically significant differences were identified between pairs of measurements using Student’s t-test (*P < 0.05, **P < 0.01, ***P < 0.001), “ns” denotes no significant difference.

Auxin, essential for grain development, has been shown to positively correlate with grain filling through 3-Indoleacetic acid (IAA) content (Chen et al., 2024). To elucidate the interaction between high temperatures and grain filling, we analyzed genes involved in IAA synthesis and transport. Following exposure to high temperatures, *ZmARF* family genes were significantly downregulated, particularly in Qi319 (**Figure 6A**; **Supplementary Figure S5**). We measured auxin levels in Zheng58 and Qi319 before and after exposure to heat stress. The results indicated a decrease in auxin content for both Zheng58 and Qi319 post-exposure, with a more pronounced reduction observed in Qi319 (**Figure 6B**). Abscisic acid (ABA) is crucial for regulating grain filling and has a positive correlation with the filling rate. We noted an increase in the expression of genes involved in ABA synthesis. Furthermore, we assessed ABA levels, observing stability in Zheng58 but a significant decrease in Qi319 after heat stress (**Figures 6C, D**).

Gibberellic acid (GA) may influence grain filling by modulating specific enzyme gene expressions, providing a material basis for this process. However, GA varieties in grains are numerous and complex. The GA3 content in Zheng58 and Qi319 grains was measured six days post-pollination, both before and after heat stress. High temperature significantly reduced GA3 levels in both Qi319 and Zheng58, with Zheng58 maintaining higher levels than Qi319 (**Figures 6E, F**). Prior research has indicated that ethylene also influences grain filling. Unable to detect ethylene directly, we measured levels of 1-aminocyclopropane-1-carboxylic acid (ACC), an ethylene precursor, before and after heat treatment. Results revealed that while ethylene levels increased after heat treatment, the rise was particularly marked in Qi319. Concurrently, we examined alterations in ethylene response genes in Zheng58 and Qi319, finding them consistent with the ACC levels. These findings suggest that increased ethylene levels post-heat treatment inhibited grain filling in Qi319 (**Figures 6G, H**). Isopentenyl transferase (IPT), tRNA dimethylallyl transferase (TRIT1), and cis-zeaxanthin O-glucosyltransferase (CISZOG) are crucial for zeatin synthesis, whereas cytokinin dehydrogenase (CKX) is essential for its breakdown (Lindner et al., 2014). Under heat stress, the expression levels of *ZmIPT*, *ZmTRIT1*, *ZmCISZOG*, and *ZmD11* in maize kernels were significantly reduced compared to control conditions, especially in Qi319 compared to Zheng58 (**Figure 6I**). After heat stress treatment, we observed a decrease in cytokinin levels in both Zheng58 and Qi319, with Qi319 showing the most significant reduction (**Figures 6J, K**). The ABA/ACC ratio serves as a crucial measure of grouting extent and positively correlates with the grouting rate. Following medium and heat stress treatment, the ABA/ACC ratio in Zheng58 increased significantly (**Figure 6L**). In summary, the differences in hormone levels between Zheng58 and Qi319 following heat stress treatment, particularly in auxin, abscisic acid, and cytokinin, primarily control grain filling.

### Heat stress restricts the transport of water and nutrients in plant stems

**Figure 7:**
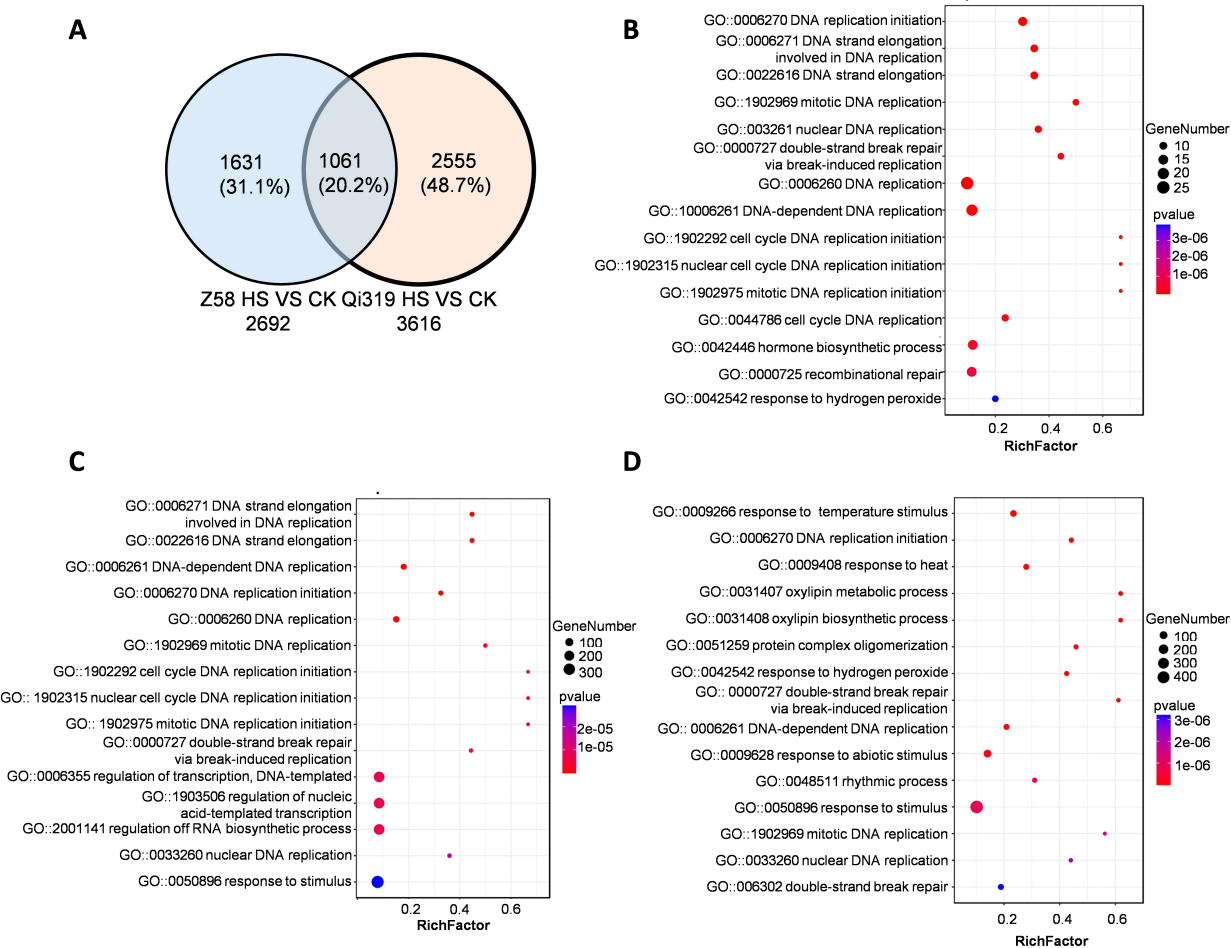
Analysis of differentially expressed genes in stems under control and high temperature conditions. **(A)** Venn diagram of DEGs in stems of Zheng58 and Qi319 under heat stress. **(B)** GO analysis of co-regulated DEGs in the stems of two maize inbred lines. The bubble size represents the number of enriched genes. **(C)** GO analysis of DEGs in stems of Zheng58 under CK and HT treatment. The bubble size represents the number of enriched genes. **(D)** GO analysis of DEGs in stems of Qi319 under CK and HT treatment. The bubble size represents the number of enriched genes.

During the grain filling stage, maize stalks play a crucial role in the transport of water, inorganic salts, and organic nutrients. This study investigates the effects of heat stress on gene expression in the stalks of two maize varieties, Zheng58 and Qi319. We exposed maize plants to the elevated temperatures during the grain-filling period and performed RNA sequencing analysis. In the stems of Zheng58 and Qi319, we identified 2,692 and 3,616 genes, respectively, with 1,061 genes common to both inbred lines. (**Figure 7A**; **Supplementary Table S5**, **S6**). Enrichment analysis of 1061 DEGs in the inbred lines under heat stress revealed significant categories including DNA replication, plant hormone regulation, and response to hydrogen peroxide (**Figure 7B**). KEGG analysis demonstrated significant enrichment of genes associated with the biosynthesis of secondary metabolites and DNA replication (**Supplementary Figure S6A**). To further elucidate the differences between Zheng58 and Qi319, we compared their transcriptomic profiles before and after heat stress. Following high temperature exposure, Zheng58 showed enrichment in genes involved in DNA replication, cell cycle, and transcriptional regulation. KEGG analysis indicated significant enrichment in metabolic biosynthesis, sucrose and fructose metabolism, and plant hormone signaling in Zheng58 after heat stress (**Figure 7C**; **Supplementary Figures S6B, C**). In contrast, Qi319 exhibited significant enrichment in genes associated with stress response, heat shock, and redox processes (**Figure 7D**; **Supplementary Figure S6D**). KEGG enrichment analysis revealed that the primary enriched genes were involved in metabolic pathways and the biosynthesis of secondary metabolites (**Supplementary Figure S6E**). Under heat stress, Qi319 shows significant enrichment of stress response genes, whereas Zheng58 predominantly enriches genes related to DNA replication. These findings suggest that Qi319 is more sensitive to heat stress, resulting increased expression of genes involved in heat stress responses.

### The differential expression of genes associated with photosynthesis and transpiration in maize ear leaves under heat stress

**Figure 8:**
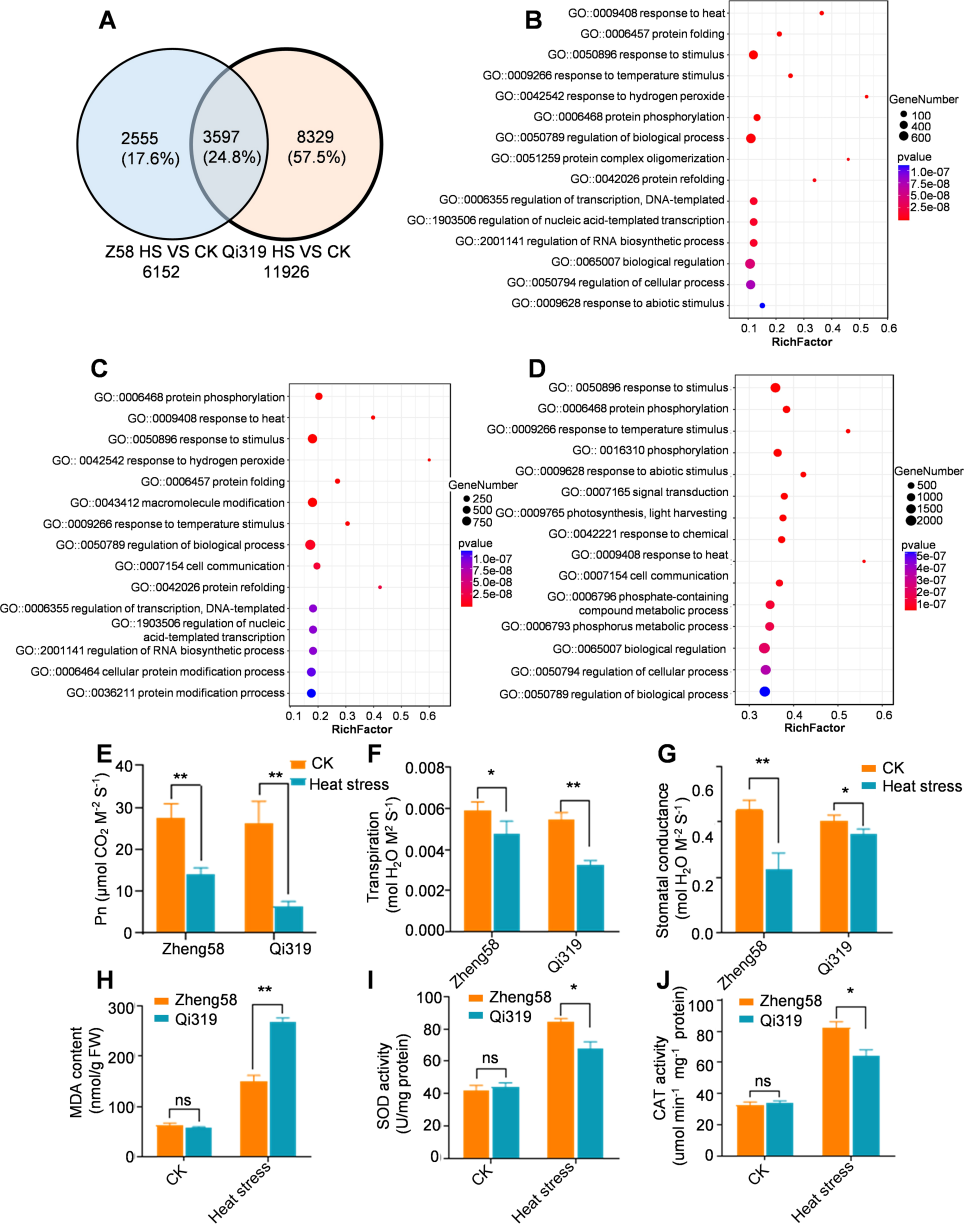
The analysis of differentially expressed genes (DEGs) in leaves under control and heat stress. **(A)** A Venn diagram illustrating the DEGs in the leaves of Zheng58 and Qi319 subjected to heat stress. **(B)** GO analysis of co-regulated DEGs in the leaves of the two maize inbred lines, where bubble size indicates the number of enriched genes. **(C)** GO analysis of DEGs in the leaves of Zheng58 following control and heat stress treatments, with bubble size representing the number of enriched genes. **(D)** GO analysis of DEGs in the leaves of Qi319 after control and heat stress treatments, where bubble size reflects the number of enriched genes. **(E)** Net photosynthetic rate (Pn) of Zheng58 and Qi319 under CK and HT treatment. Data are presented as mean ± SE. Statistical significance was assessed using a two-tailed paired t-test, with asterisks indicating significant differences as follows: **P < 0.01. **(F)** Transpiration rates were measured under normal conditions and following heat stress treatment, with data presented as mean ± SE. Statistical significance was evaluated using a two-tailed paired t-test, with asterisks indicating significant differences as follows: *P < 0.05, **P < 0.01. **(G)** Stomatal conductance measurements for Zheng58 and Qi319 under normal conditions and after heat stress treatment are presented as mean ± SE. Statistical significance was determined using a two-tailed paired t-test, with asterisks denoting significant differences as follows: **P < 0.01, and ‘ns’ indicating no significant difference. **(H-J)** This study investigates the levels of malondialdehyde (MDA), superoxide dismutase (SOD), and catalase (CAT) in the leaves of Zheng58 and Qi319, both under control conditions and following heat stress treatment. The data are presented as mean ± SE. Statistical significance was evaluated using a two-tailed paired t-test, with asterisks denoting significant differences as follows: *P < 0.05, **P < 0.01, ***P < 0.001. The notation ‘ns’ indicates no significant difference.

Maize leaves, the primary site of photosynthesis, are crucial for producing organic matter essential for plant growth and for regulating responses to abiotic stresses through transpiration. To explore the differences in leaf regulatory genes between Zheng58 and Qi319 under high temperatures, we conducted RNA sequencing on their panicle leaves following 7 days of stress treatment. Under heat stress, compared to the control, 6152 genes in Zheng58 and 11926 genes in Qi319 were altered, with 3597 DEGs common to both inbred lines (**Figure 8A**; **Supplementary Tables S7**, **S8**). GO enrichment analysis DEGs revealed that the top five responses were to heat, protein folding, stimulation, high temperature, and hydrogen peroxide (**Figure 8B**). Furthermore, analysis from the Kyoto Encyclopedia of Genes and Genomes indicated significant enrichment of genes associated with endoplasmic reticulum protein processing, plant-environment interaction, and the MAPK signaling pathway (**Supplementary Figure S7A**). The results demonstrate that under heat stress, Zheng58 and Qi319 co-regulate genes involved in stress response and endoplasmic reticulum protein processing.

To identify genes differentially regulated by Zheng58 and Qi319 under heat stress, we conducted separate analyses for each inbred line. In the inbred line Zheng58, heat stress upregulated genes associated with response activation, biological process regulation, and polymer modification (**Figure 8C**; **Supplementary Figures S7B, C**). In Qi319, genes associated with abiotic stress response, light signaling, and biological regulation were significantly enriched (**Figure 8D**; **Supplementary Figure S7E**). This suggests that genes related to photosynthetic reactions are differentially expressed in Zheng58 and Qi319 after heat stress. To confirm this finding, we employed qRT-PCR to assess the expression of photosystem-related genes. Consistent with RNA-seq data, under control conditions, expression of the light-harvesting complex genes (*ZmLHCA2*, *ZmLHCA3*, *ZmLHCA4*, *ZmLHCB1*, *ZmLHCB4*, *ZmLHCB5*, and *ZmLHCB6*) and photosystem I/II subunits (*ZmpsaC*, *ZmpsaH*, *ZmpsaO*, *Zmpsb27*, *Zmpsb28*, *ZmpsbB*, *ZmpsbQ*, and *ZmpsbW*) was downregulated in both inbred lines due to heat stress. The reduction in expression was less pronounced in Zheng58 compared to Qi319 (**Supplementary Figure S8A**). We tested the photosynthesis of the Zheng58 and Qi319 inbred lines both before and after heat stress treatment. The results indicated that the net photosynthetic rate (Pn) and intercellular CO_2_ concentration (Ci) in both Zheng58 and Qi319 were significantly reduced following heat stress treatment, with a more pronounced reduction observed in Qi319 (**Figure 8E**; **Supplementary Figures S8B**).

Heat stress also decreased the transpiration rates of Zheng58 and Qi319 panicle leaves by 19.5% and 40.3%, respectively (**Figure 8F**). We also measured the water content and stomatal conductance of Zheng58 and Qi319 before and after heat stress and found that the stomatal conductance of Zheng58 was significantly lower than that of Qi319 after heat stress (**Figure 8G**; **Supplementary Figure S8C**). The antioxidant defense system plays a key role in alleviating oxidative damage, among which enzymes such as superoxide dismutase (SOD) and catalase (CAT) play a vital role in clearing ROS under heat stress (Liu et al., 2022). We measured the contents of Zheng58 and Qi319 before and after heat stress treatment and found that after heat stress treatment, the SOD and CAT contents in Qi319 were significantly lower than those in Zheng58, while the malondialdehyde (MDA) content was higher than that in Zheng58 (**Figures 8H–J**). The MDA content is an indicator of oxidative damage during plant stress. The above results indicate that Qi319 oxidative damage increases under heat stress and is sensitive to heat stress. In summary the decline in photosynthesis and transpiration in Qi319 due to heat stress significantly impacts grain filling.

## Discussion

### Multiple factors affect grain filling under heat stress

**Figure 9:**
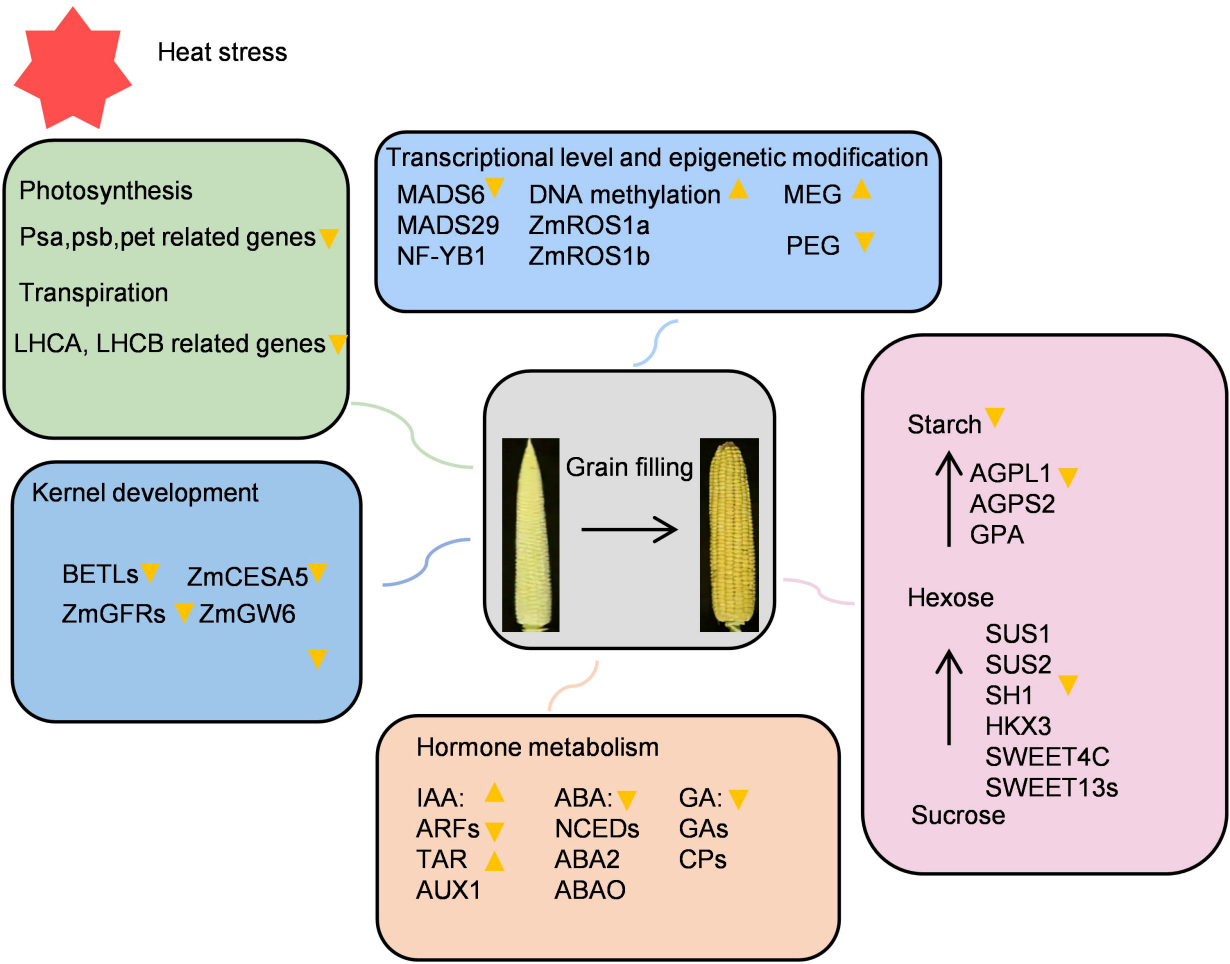
Effects of heat stress on maize grain filling period.

Heat stress affects yield through multiple factors (**Figure 9**). Short-term heat stress before and after flowering will interfere with flowering and reduce the amount of pollen shedding and its vitality (Jagadish, 2020; Chen et al., 2024). The number and activity of pollen are important factors limiting yield. Recent studies have shown that increasing PSW can improve yield (Liu et al., 2023). Previous studies have shown that the effects of heat stress on male reproductive organs of maize are expected to be greater than those on females because the tassels are directly exposed to sunlight and affected by heat waves (Lizaso et al., 2018). However, this study found that the pollen quantity of the inbred line Zheng58 was slightly lower than that of Qi319 under normal conditions or heat stress conditions, and there was no significant difference in pollen activity (**Figure 2A**). This indicates that the effect of heat stress on Zheng58 and Qi319 is determined by maternal factors.

The impact of high temperature on the mother plant also involves multiple aspects. First, during the grain filling period, high temperature can accelerate grain filling and terminate maize endosperm development early. This delays the maturation of the grain, resulting in reduced starch accumulation in the grain, which ultimately reduces grain weight (Guo et al., 2021). Heat stress causes abnormal hormone homeostasis in the plant body, and differences in photosynthesis, transpiration, and respiration are also the reasons for the reduction in yield (Huang et al., 2016; Doll et al., 2017; Wang et al., 2024). Heat stress during the early stages of maize development adversely affects grain filling, reducing both its rate and weight, and may even completely halt the process (Wu et al., 2024b). However, its internal regulatory network and molecular mechanism are still unclear. this study utilized the heat-tolerant inbred line Zheng58 alongside the heat-sensitive inbred line Qi319 employed transcriptome analysis to investigate the impact of high temperatures during the grain filling period on the growth and development of maize grains across various heat-resistant varieties. The research specifically focused on the effects on endosperm cell development, sucrose and starch metabolism, hormone levels, photosynthesis, transpiration, and epigenetic changes. Heat stress disrupts sugar metabolism, impairing both the conversion of sucrose to glucose and the synthesis of starch, which adversely affects grain filling. Furthermore, elevated temperatures reduce the levels of hormones such as auxin, abscisic acid, and zeaxanthin, which further impede the grain filling process. High temperatures also compromise the transportation of water and inorganic salts in maize stalks, negatively influencing grain filling. Additionally, elevated temperatures diminish photosynthetic activity and transpiration rates, delaying the synthesis of essential substances required for grain filling. Lastly, heat stress induces abnormal gene expression, which alters grain morphology and increases the proportion of deformed grains (**Supplementary Figure S8**). Overall, our multi-omics analysis of maize kernel development under heat stress provides insights into the process of maize yield formation under heat stress conditions.

### Heat stress affects endosperm development

Abiotic stress in the early stages of grain development disrupts endosperm cell division (Barnabás et al., 2008), resulting in a smaller endosperm (Begcy and Walia, 2015), which ultimately severely affects maize yield (Begcy et al., 2019). The significant impact of abiotic stress on seed development led to numerous deformities and cavities within the seeds, suggesting that embryo development was compromised and grain filling was impeded (Schmidt et al., 2020). Maize endosperm development begins with the fertilization of the central cell, followed by cellularization, mitosis, and rapid differentiation into four distinct cell types: the basal endosperm transfer layer (BETL), aleurone layer (AL), starchy endosperm (SE), and embryonic surrounding region (ESR) (Dai et al., 2021; Zheng, 2022). To investigate the impact of heat stress on maize kernel filling, we employed qRT-PCR to measure the expression of specific genes within the aforementioned cell types and evaluate their response to heat stress. The BETL primarily facilitates nutrient absorption and transport. Currently, the MYB-related protein ZmMRP-1 is the only transcription factor known to regulate its development. ZmMRP-1 controls the expression of several endosperm basal transfer genes, including *ZmTCRR-1*, *ZmTCRR-2*, *ZmMEG1*, *ZmBETL-1*, *ZmBETL-2*, *ZmBETL-9*, and *ZmBETL-10* (Royo et al., 2009). To assess the response of these genes to heat stress, we conducted transcriptome analysis and qRT-PCR. Our findings revealed that these genes were significantly down regulated following heat stress, suggesting that elevated temperatures markedly inhibit the activity of the BETL (**Supplementary Figure S9A**). NAKED ENDOSPERM1 (*NKD1*) and *NKD2* are duplicated INDETERMINATE DOMAIN (*IDD*) transcription factors that play a crucial role in maize endosperm development and are specifically expressed in the AL (Wu et al., 2024a). Genes with AL-specific expression are significantly upregulated following heat stress treatment. The transcription factor O2 regulates the expression of genes that are specifically expressed in the ESR during various stages of embryonic differentiation. The genes *ZmAE1*, *ZmAE3*, *ZmEsr1*, *ZmEsr2*, *ZmEsr3*, and *ZmEsr6* are specifically expressed in the ESR (Wu et al., 2024a). We assessed gene expression levels following heat stress treatment. The results indicate a significant increase in the expression of ESR-specifically expressed genes after exposure to heat stress (**Supplementary Figure S9B**). During endosperm cell development, *ZmCDK*, a gene that regulates the cell cycle, is specifically expressed in endosperm cells. Our findings revealed that heat stress treatment significantly reduced the expression of cell cycle genes. This suggests that elevated temperatures decrease the abundance of genes expressed in the BETL and inhibit endosperm cell division, ultimately leading to insufficient grain filling. Concurrently, heat stress induced abnormal expression of genes associated with grain morphology, resulting in grain deformity. This observation further corroborates the underlying cause of grain deformity under heat stress and aligns with previous findings on grain deformity in response to abiotic stress (Schmidt et al., 2020).

### Heat stress influences the intricate regulatory network of hormones and endosperm development during the grain filling process

Seven days post-pollination, we observed that elevated temperatures accelerated the kernel filling rate but reduced the overall kernel filling abundance. The TKW decreased following exposure to heat stress, which is closely associated with all aspects of the grain filling process, particularly during the rapid filling period. Notably, the reduction in starch synthesis is the most significant manifestation of this effect. In the starch synthesis pathway, hexokinase plays a critical role in converting hexose into starch by regulating the conversion of glucose to glucose-6-phosphate, while SUS facilitates the conversion of sucrose into hexose (Vanderwall and Gendron, 2023). In this study, heat stress downregulated the expression levels of *ZmSUS* and decreased its activity, particularly in the heat-sensitive inbred Qi319. In contrast, the heat-tolerant inbred Zheng58 exhibited higher total soluble sugar content and stable sucrose accumulation across various temperature treatments. The increase in sugar content enhances osmotic regulation and mitigates severe heat damage. Previous research has indicated that the decomposition and resynthesis of sucrose can improve carbohydrate partitioning, thereby enhancing plant tolerance to abrupt environmental changes. The equilibrium conversion between sucrose and hexose is essential for plant adaptation to heat stress, with HK and SUS playing critical roles in this process. However, the interconversion of sucrose and hexose in maize is regulated by multiple pathways, including alterations in enzyme activities, hormonal regulation, and stress responses. Notably, prior studies have demonstrated that IAA regulates the activity of starch-sucrose-related enzymes, including sucrase, SUS, starch synthase (StSase), and starch branching enzyme (SBE) (Zhao et al., 2022). Heat stress results in an increase in the activity of enzymes related to sucrase and sucrose synthase, while simultaneously causing a decrease in auxin levels. Moreover, auxin regulates the activity of both sucrase and sucrose synthase, indicating that under heat stress, plants utilize multiple pathways to modulate the activity of stress-related enzymes. This adjustment ensures adequate sucrose content, which serves as an osmotic regulating substance, aiding plants in maintaining cellular osmotic balance and promoting the synthesis of metabolites to heat stress (Li et al., 2022a).

Plant hormone biosynthesis and signaling play a crucial role in influencing plant tolerance to heat stress. In this study, transcriptome analyses and hormone assays revealed that heat stress significantly downregulated the expression of the gene encoding IPT, which catalyzes the rate-limiting step in cytokinin synthesis in two inbred line species. Specifically, heat stress reduced the transcript levels of *ZmIPT*, *ZmTRIT1*, and *ZmCISZOG*, all of which are involved in cytokinin biosynthesis. We observed a significant reduction in the expression levels of these related genes after high-temperature treatment, this effect was particularly pronounced in the heat-sensitive mutant Qi319. Additionally, although the content of zeaxanthin decreased after heat stress, the reduction was more marked in Zheng58. GAs play a positive role in regulating grain filling by promoting the enlargement of maternal cells, increasing capacity, and enhancing photosynthesis. The exogenous application of GA3 has the potential to transform weak grains into strong ones. Additionally, GAs can stimulate the synthesis of IAA and inhibit the activity of StSase, thereby reducing starch synthesis and accumulation. ABA and ethylene facilitate the filling process in the early stages and accelerate the completion of filling in the later stages (Cui et al., 2020; Lu et al., 2022). After assessing their levels after heat stress, we observed significant increases in ABA and the ACC. These findings are critical for understanding how plants endure heat stress and efficiently complete their reproductive cycles. Under conditions of heat stress, plant hormones play various roles in regulating grain filling. The mechanisms that balance the grain filling rate and abundance create a complex regulatory network.

### Heat stress induce high temperature memory by influencing epigenetic modifications

These modifications are essential for the environmental adaptability of plants. Key processes such as DNA methylation, histone acetylation, histone post-translational modifications, and chromatin remodeling play critical roles in the response to abiotic stresses. DNA demethylation is primarily governed by the demethylase gene *ZmROS1*. Two important genes, *ZmROS1a* and *ZmROS1b*, which function as DNA demethylases, are involved in regulating the development of grain endosperm (Xu et al., 2022). To evaluate the response to elevated temperatures, we employed qRT-PCR to measure methyltransferase levels following heat treatment. The results indicated that, after exposure to heat stress, *ZmROS1* expression was significantly downregulated in Zheng58, whereas Qi319 exhibited no significant change (**Supplementary Figure S10A**). In maize, adenosine methyltransferase (*ZmMTA*) interacts with DNA methylation 1 (*ZmDDM1*) to regulate embryogenesis and endosperm development. The results indicated a slight reduction following heat stress treatment (**Supplementary Figure S10A**). These findings suggest that heat stress treatment may elevate DNA methylation levels in maize kernels, thereby enhancing resistance to heat stress. Research indicates that DNA methylation in plant genomes undergoes changes in response to both biotic and abiotic stresses, and these alterations can be inherited by future generations. Imprinted genes play a crucial role in regulating grain development. Whole-genome surveys in maize have identified hundreds of imprinted genes within the endosperm. The majority of these are maternally expressed imprinted genes (MEGs), while a smaller subset consists of paternally expressed imprinted genes (PEGs). Imprinted genes are regulated epigenetically through mechanisms such as DNA methylation, histone modification, long noncoding RNAs, and higher-order DNA structures. The expression of imprinted genes is influenced by parental effects. We conducted a quantitative analysis to assess the response of specific maternal imprinted genes in maize to heat stress. The results indicated that, following heat stress treatment, most maternal imprinted genes were downregulated (**Supplementary Figures S10B, C**).

Previous studies have demonstrated that both *ZmFIE1* and *ZmFIE2* are maternally imprinted genes. The overexpression of *OsFIE1* leads to enlarged grain development; however, its expression is significantly suppressed under heat stress (Dhatt et al., 2021). This observation aligns with our findings, further suggesting that heat stress has a considerable impact on the expression of imprinted genes. Regarding plant high-temperature memory, brief exposure to elevated temperatures can enable plants to establish a memory of heat stress, thereby enhancing their ability to cope with future severe heat conditions. The induction of epigenetic changes facilitates plants’ rapid adaptation to short-term environmental fluctuations (Chen et al., 2016; Wibowo et al., 2016; Malik and Zhao, 2022). Recent reports indicate that numerous genes in *Arabidopsis*, including *ZmHsf-01*, *ZmHsf-15*, *ZmHsfA3*, *ZmUVH6*, *ZmHO-1*, *ZmGSL-1*, *ZmCycD2;1*, and *ZmCycD5;2*, are regulated by acetylation in response to heat stress (**Supplementary Figure S10A**). Analysis of the expression of acetylation-related genes has revealed a significant downregulation following heat stress, particularly in the cultivar Zheng58. This finding suggests that acetylation plays a crucial role in regulating grain filling in plants. Plant domestication is a complex process that involves the regulation of physiological, genetic, and protein levels, as well as various signaling pathways, with epigenetics being a key factor in this process. Grain filling serves as a vital link between the mother plant and the next generation. Therefore, analyzing the specific mechanisms by which stress impacts grain filling is significant for understanding plant domestication. Identifying key genes essential for high-temperature acclimation and employing genetic engineering techniques can enhance crop resilience to adverse stresses, ultimately boosting yields.

## Conclusion

Utilizing heat-resistant maize varieties is a cost-effective and efficient strategy to mitigate heat stress. The development and promotion of varieties that are resistant to both heat and drought represent the most effective approach. Heat resistance enables crops to adapt to heat stress. Current research on heat stress in our country is still in its nascent stages, with a limited understanding of its relationship with key factors. Consequently, the impact of elevated temperatures on maize filling is still undergoing preliminary investigation, and research into temperature-related damage is inherently complex. Heat resistance varies among different maize varieties, growth stages, and environmental conditions. It is essential to create targeted strategies for addressing various stress factors and growth stages of maize to optimize adaptation to climate change and ensure high yields, which is critical for both scientific research and practical cultivation. Furthermore, by examining the differing performances of the high-temperature resistant inbred line Zheng58 and the high-temperature sensitive inbred line Qi319 under elevated temperatures, we can provide theoretical support for the directional implementation of molecular design breeding aimed at cultivating new varieties that exhibit high yields, rapid filling, and resistance to heat stress.

## Data Availability

The RNA-seq original data are deposited in the National Genomics Data Center (NGDC) with the GSA accession number CRA021527.

## Supplementary material

Supplementary Figure 1 Cluster analysis of seed setting rates of different maize inbred Lines.

Supplementary Figure 2 Relative expression of pollen viability and silk-specifically expressed genes.

Supplementary Figure 3 Enrichment analysis of differentially expressed genes in Zheng58 under normal conditions and following heat stress treatment.

Supplementary Figure 4 Enrichment analysis of differentially expressed genes in seeds of Zheng58 and Qi319 under heat stress.

Supplementary Figure 5 The expression of the *ZmARF* family.

Supplementary Figure 6 Enrichment analysis of differentially expressed genes in stems of Zheng58 and Qi319 under heat stress.

Supplementary Figure 7 Enrichment analysis of differentially expressed genes in leaves of Zheng58 and Qi319 under heat stress.

Supplementary Figure 8 The relative expression of light-harvesting complex genes.

Supplementary Figure 9 The relative expression of basal endosperm transfer genes and embryonic surrounding region ESR-specifically expressed genes.

Supplementary Figure 10 Epigenetic modifications are involved in the effects of heat stress on grain filling.
