# Supplementary material for: The molecular mechanism by which heat stress during the grain filling period inhibits maize grain filling and reduces yield
Source: Front Plant Sci. 2025 Jan 17;15:1533527. doi: 10.3389/fpls.2024.1533527 (PMC11782181; doi:10.3389/fpls.2024.1533527)
Supplement: Supplementary Figure 1 — Cluster analysis of seed setting rates of different maize inbred Lines. [file DataSheet1.zip › FigS.docx]

**Figure S1:** Cluster analysis of seed setting rates of different maize inbred Lines.

**Figure S2:** Relative expression of pollen viability and silk-specifically expressed genes.

**Figure S3:** Enrichment analysis of differentially expressed genes in Zheng58 under normal conditions and following heat stress treatment.

**Figure S4:** Enrichment analysis of differentially expressed genes in seeds of Zheng58 and Qi319 under heat stress.

**Figure S5:** The expression of the *ZmARF* family.

**Figure S6:** Enrichment analysis of differentially expressed genes in stems of Zheng58 and Qi319 under heat stress.

**Figure S7:** Enrichment analysis of differentially expressed genes in leaves of Zheng58 and Qi319 under heat stress.

**Figure S8:** The relative expression of light-harvesting complex genes.


**Figure S9:** The relative expression of basal endosperm transfer genes and embryonic surrounding region ESR-specifically expressed genes.

**Figure S10:** Epigenetic modifications are involved in the effects of heat stress on grain filling.
